# Linking cognitive reserve to neuropsychological outcomes and resting-state frequency bands in healthy aging

**DOI:** 10.3389/fnagi.2025.1540168

**Published:** 2025-03-17

**Authors:** Vanesa Perez, Vanesa Hidalgo, Alicia Salvador

**Affiliations:** ^1^Laboratory of Social Cognitive Neuroscience, Department of Psychobiology, IDOCAL, University of Valencia, Valencia, Spain; ^2^Research Group of Psychology and Quality of Life (PsiCal), Valencian International University, Valencia, Spain; ^3^Department of Psychology and Sociology, Area of Psychobiology, University of Zaragoza, Teruel, Spain; ^4^Spanish National Network for Research in Mental Health CIBERSAM, Madrid, Spain

**Keywords:** electroencephalography, frequency bands, cognitive reserve, healthy aging, neuropsychological assessment

## Abstract

**Introduction:**

As the proportion of older people has surged in the past 100 years, healthy aging has emerged as a crucial topic in neuroscience research. This study aimed to investigate the spectral power of EEG frequency bands during resting-state in older people with high and low cognitive reserve (CR).

**Methods:**

To do so, 74 healthy older people (55–74 years old) were recruited and divided into two groups based on their level of CR: high CR (*n* = 41; 21 men and 20 women) and low CR (*n* = 33; 15 men and 18 women). Both groups participated in a cognitive assessment and 3 min of EEG recording under resting-state conditions with eyes open (EO) and eyes closed (EC). EEG power was analyzed across four frequency bands: delta (0.1– < 4 Hz), theta (4– < 8 Hz), alpha1 (8–10 Hz), alpha2 (10–12), and beta (14–30 Hz), focusing on five cortical regions of interest.

**Results:**

Neuropsychological tests did not reveal significant differences between the two groups on most of the cognitive measures. However, the EEG analysis showed that individuals with high CR exhibited lower spectral power in the theta and delta frequency bands across different brain regions, compared to those with low CR.

**Discussion:**

These findings suggest that individuals with high CR tend to function more efficiently, relying on fewer neural resources to sustain cognitive performance. In contrast, those with low CR may engage compensatory neural mechanisms, as indicated by increased spectral power while resting, conceivably reflecting the brain’s effort to preserve cognitive function.

## 1 Introduction

With the rapid aging of the global population, public health challenges such as cognitive decline and dementia have emerged as critical priorities (World Health Organization [WHO], 2023). However, cognitive changes in older adults vary widely. Whereas some individuals experience a rapid decline, others preserve their cognitive abilities, exemplifying successful aging (Stern et al., 2019). Furthermore, this heterogeneity in aging and its various manifestations, along with the delayed onset of symptoms in individuals with neurodegenerative diseases, has led to the hypothesis of an underlying cognitive reserve (CR) that mitigates the expected cognitive decline (Pappalettera et al., 2024).

Two models have been proposed to explain the underlying reserve mechanism. From a structural perspective, the passive or brain reserve hypothesis explains clinical and cognitive differences by suggesting that larger brains (with greater numbers of neurons and synapses) are more resilient to pathology, thus preventing the clinical or behavioral manifestations of dementia (Barulli and Stern, 2013). According to this concept, the subject’s brain passively experiences the pathology and, depending on each person’s characteristics, will manifest it in a more or less lasting way. Conversely, from a functional perspective, the CR hypothesis posits that lifelong experiences (intellectual, occupational, physical, and social activities) enable individuals to better endure the consequences of neurological diseases (Stern and Barulli, 2019). CR refers to the brain’s capacity to tolerate age-related changes and disease-related pathologies without exhibiting evident clinical symptoms (Stern, 2009). This active and dynamic concept of CR allows the brain to adapt to deteriorating conditions by employing cognitive resources to compensate for deficits. It also helps to explain individual differences in the susceptibility to cognitive, functional, or clinical decline due to aging or brain disease (Stern et al., 2020).

Several studies have investigated the variations in cognitive performance between individuals with high and low levels of CR. These studies have demonstrated that CR plays a crucial role in modulating brain activity and efficiency while performing cognitive tasks such as memory, verbal memory, working memory, attention, executive function, and orientation (Babiloni et al., 2021; Feldberg et al., 2024; Lavrencic et al., 2018; Montoliu et al., 2023), as well as in delaying the onset of cognitive decline related to diseases (Pettigrew and Soldan, 2019). However, it is important to note that no significant differences have been observed in other cognitive domains, such as emotional perception, processing speed, or motor performance, between individuals with high and low CR (Lavrencic et al., 2018).

Despite these findings, the neural basis underlying CR is not yet fully understood. Analyzing electroencephalographic (EEG) rhythms in resting subjects is a valuable, low-cost neurophysiological approach that can provide insight into the mechanisms supporting CR. Resting-state brain activity, measured when individuals are awake and relaxed, offers a unique opportunity to assess the baseline neural function without the influence of external cognitive tasks (Babiloni et al., 2020a). Research on the influence of CR on resting-state brain function has primarily focused on patients with Alzheimer’s disease (Bastin et al., 2012). However, studies exploring the electrophysiological basis of CR in healthy older people remain limited.

An EEG study by Babiloni et al. (2020b) found a significant relationship between estimated CR and EEG activity in older people with subjective memory complaints (SMCs), showing that those with a positive amyloid status and higher CR (measured by education and occupation) exhibited higher temporal alpha amplitudes and lower posterior alpha amplitudes, compared to those with lower CR. Similarly, Babiloni et al. (2021) analyzed resting-state EEG data from healthy older people and people with mild cognitive impairment (MCI), using education levels as a proxy for CR. Their results indicated that healthy older people with high CR showed greater alpha EEG activity, whereas those with MCI and high CR had lower alpha EEG activity than their low CR counterparts. The authors suggested that high CR modulates resting-state EEG alpha rhythms, offering potential neuroprotection in healthy people and compensation in those with MCI. Additionally, neuroimaging studies have shown that older people with high CR exhibit reduced cerebral metabolic activity in the temporoparietal cortex during the resting state. Consistent with these findings, our previous EEG study revealed that higher CR was associated with a slowing of the alpha peak in older people (Perez et al., 2024).

Expanding on the potential applications of EEG, a review by Šneidere et al. (2020) highlights that resting-state EEG may serve as a screening tool for early cognitive decline rather than a definitive biomarker. However, findings related to CR in resting-state EEG studies have been inconsistent, with some reporting no significant relationship and others identifying differences in hemispheric coherence across CR levels (Choi et al., 2019; Fleck et al., 2017; Šneidere et al., 2020). Notably, Fleck et al. (2017) observed that high levels of CR in older people are associated with increased right-hemisphere coherence, particularly in the theta and high alpha frequency bands, which highlight the potential of CR to promote efficiency in connectivity during aging. Additionally, studies analyzing spectral power have reported specific associations between CR and oscillatory activity. For instance, Ferrari-Díaz et al. (2022) found that leisure activities, often considered a dynamic proxy for CR, were positively associated with increased posterior alpha2 power, reinforcing the role of CR in modulating resting-state EEG activity through compensatory mechanisms.

Building on existing literature, the present study aimed to explore the impact of CR on neuropsychological performance and resting-state EEG spectral power across different frequency bands in healthy older adults. The first hypothesis posited that individuals with higher CR would demonstrate better performance on neuropsychological assessments that evaluated various cognitive domains (Babiloni et al., 2021; Feldberg et al., 2024; Montoliu et al., 2023). Additionally, we expected that older people with high CR to exhibit higher spectral power in the alpha band and lower spectral power in the theta bands compared to those with low CR (Babiloni et al., 2020b; Balart-Sánchez et al., 2021). Regarding delta activity, previous findings show contradictory results. On the one hand, using verbal intelligence as a proxy for CR, Ferrari-Díaz et al. (2022) linked higher verbal intelligence to increased delta power. On the other hand, using physical activity as a proxy for CR, Sánchez-López et al. (2018) associated lower delta power in certain regions with greater physical activity. Due to these inconsistencies, a hypothesis cannot be drawn. Therefore, further investigation is needed to determine whether delta power is consistently associated with CR. No significant differences in beta power are expected between high and low CR groups, as reported by Sánchez-López et al. (2018).

## 2 Materials and methods

### 2.1 Participants

For this study, the sample recruited contained 82 participants. A power analysis conducted using G*Power determined that this sample size would be sufficient to detect small to medium effect sizes (*f* = 0.145) in a repeated-measures ANOVA. This calculation was based on a power of 0.90, a Bonferroni-adjusted α = 0.005, and previous research findings. However, due to technical issues, the final sample consisted of seventy-four healthy older adults enrolled in this study. The older people were recruited from La Nau Gran, a study program for people over 55 years old at the University of Valencia (Spain). The exclusion criteria included: unimpaired objective cognition (Mini-Mental State Examination [MMSE] score above 27), smoking more than 10 cigarettes per day, history of alcohol or drug abuse, recent surgery under general anesthesia (within the past year), visual or hearing impairments, or any illness affecting the nervous system. Participants with neurological or psychiatric disorders were also excluded. Additionally, individuals were excluded if they were taking medications that could affect cognitive or emotional function, psychoactive substances, or beta-blockers, or if they had experienced a significant stressful event in the past 6 months. Only right-handed participants (determined by the Edinburgh Handedness Inventory; Oldfield, 1971) were included. Eligible participants were contacted by telephone and invited to attend two sessions at the Laboratory of Social Cognitive Neuroscience at the University of Valencia (Spain).

Participants were divided into two different groups based on their scores on the CR Questionnaire. Participants with scores equal to or above 16 were classified into the high CR group, whereas those with scores below 16 were placed in the low CR group. The median score of 16 on the CR scale was used as the threshold in order to ensure an equal distribution of participants across the groups. This cutoff point is commonly utilized because it makes it possible to create balanced groups, which facilitates statistical comparisons and increases the power to detect differences between high and low CR individuals. A similar approach was employed by Di Tella et al. (2023), who also used the median to divide the sample into high and low CR groups.

### 2.2 Procedure

Each participant was tested individually. Upon arrival at the laboratory, the experimenter made sure that participants had adhered to the pre-session guidelines: avoid strenuous physical activity and maintain their usual sleep patterns the night before, abstain from alcohol and stimulants (such as caffeine, cola, tea, or chocolate), and refrain from eating or smoking for at least 2 h prior to the session. Participants were also instructed to drink only water.

Participants completed two experimental sessions on consecutive days. Half of the participants attended in the morning (between 10:00 and 12:00), and the other half attended in the afternoon (between 15:00 and 19:00). To minimize potential fatigue effects, participants were randomly assigned to either morning or afternoon sessions to ensure an even distribution of potential time-of-day effects. Additionally, all participants were given adequate breaks during the sessions. On the first day, participants completed various questionnaires, including the CR tests. On the second day, the resting-state EEG session took place. During this session, participants sat comfortably in a quiet, softly lit room while EEG activity was recorded. First, participants kept their eyes open (EO) for 3 min, followed by another 3 min with their eyes closed (EC). They were instructed to remain still, relax their muscles, avoid voluntary movements, and not speak. An experimenter monitored the EEG traces in real time and alerted participants if there were signs of drowsiness or behavioral changes. All the participants followed these instructions without any difficulty.

#### 2.2.1 Cognitive reserve (CR)

We assessed each participant’s CR using the Cognitive Reserve Questionnaire, which considers both formal (education and work) and leisure activities (Rami et al., 2011). It consists of eight items, each with between three and six response options, that assess different facets of intellectual activity, including education, parental education level, their main lifetime occupation, musical training, proficiency in languages, and participation in training courses. Additionally, it examines the approximate frequency of cognitively stimulating lifestyle activities, such as reading habits and playing intellectual games. The maximum score is 25, with higher scores indicating a greater CR. A score of 6 or less is considered the lowest CR. The scale’s reliability in this sample was estimated using the categorical omega coefficient ωNL = 0.731 (Hayes and Coutts, 2020).

#### 2.2.2 Neuropsychological assessment

The neuropsychological battery used in this study included the MMSE, a widely used screening test that provides a brief measure of cognitive function (Lobo et al., 1979). Attention and working memory were evaluated using the Digit Span Task from the Wechsler Memory Scale (Wechsler, 1997). This task is designed to evaluate working memory by requiring participants to listen to a series of numbers and then repeat them in the same order (forward) and in reverse order (backward). The sequences begin at level 2 and can progress to level 8. Participants are given two attempts for each sequence length, and if they successfully repeat one of the sequences, they proceed to the next level.

Additionally, attention-switching was examined using the Trail-Making Test (TMT), which consists of two parts: TMT-A and TMT-B (Reitan, 1992). TMT-A involves connecting circles numbered from 1 to 25 in ascending order as quickly as possible. TMT-B requires participants to connect circles in a sequence that alternates between numbers (1 to 13) and letters (A to L). The outcomes measured consisted of the total time taken to complete each part (TMT-A and TMT-B).

Additionally, the Rey Figure Test was used to assess visual-spatial construction and memory. Participants were timed on two components: (i) the copying time, which measures the ability to accurately reproduce a complex figure and reflects visual-spatial construction skills; and (ii) the delayed recall time, which evaluates the ability to recall and reproduce the figure from memory after a delay, providing insight into visual memory retention (Rey, 1941).

Phonological and semantic fluency were assessed by asking participants to generate as many words as possible in each category in 60 s. For phonological fluency, participants produced words starting with the letters F, A, and S, whereas for semantic fluency, they generated words related to the category of animals. Only correct, unique responses were scored, excluding intrusions, repetitions, and variations within the same species. The tests were administered according to the procedures outlined in the Barcelona Test (Peña-Casanova, 1991).

Visuo-spatial working memory was evaluated using the Automated Working Memory Assessment (AWMA; Alloway et al., 2008). The Dot Matrix Forward subtest required participants to identify red dots in their original sequence to measure attention and memory span. The Dot Matrix Backward subtest involved pointing to boxes in reverse order to evaluate the executive function of working memory. The outcomes recorded were: (i) Dot Matrix-Forward, indicating the number of correct trials in the original sequence; and (ii) Dot Matrix-Backward, reflecting the number of correct trials in reverse order. Scores ranged from 2 to 8, and participants were allowed two attempts per sequence length. Successful performance led to progression to the next sequence.

Verbal memory was assessed using the Spanish version of the Free and Cued Selective Reminding Test (FCSRT; Palomo et al., 2013). On this test, a list of 16 words is initially presented, followed by a distractor task that involves subtracting numbers for 20 s. Participants then engage in free recall of the list of words for 90 s. Facilitated recall provides prompting for words not recalled during the free recall. The test includes three trials, yielding five indices: (i) free recall on the first trial (max 16 points); (ii) total free recall (sum of all three trials, max 48 points); (iii) total recall (sum of free and facilitated recall, max 48 points); (iv) free delayed recall (sum of free recall after a delay, max 16 points); and (v) total delayed recall (sum of free and facilitated deferred recall, max 16 points).

#### 2.2.3 Resting state EEG recording

EEG recording was obtained using an EEG cap (Easycap, Falk Minow, Munich, Germany) with 29 electrode positions aligned to the 10–20 System (Fp1, Fpz, Fp2, AFz, F7, F3, Fz, F4, F8, FCz, M1, T3, C3, Cz, C4, T4, M2, T5, P3, Pz, P4, T6, O1, Oz, and O2). The ground electrode was positioned at AFz, and the data were initially referenced to FCz. To ensure optimal signal quality, impedance at each electrode site was maintained below 5 kΩ by using a high-viscosity electrolyte gel (SUPER-VISC High Viscosity Electrolyte-Gel, EasyCap, Brain Products GmbH). The EEG signals were bandpass filtered between 0.3–100 Hz, and data were sampled at a rate of 500 Hz. After filtering, the data were re-referenced to the average of all the remaining electrodes. Vertical and horizontal eye movements were monitored through additional electrodes positioned around the eyes (VEOG−, VEOG+, HEOG-, HEOG+). Blink-related artifacts were identified and removed using Independent Component Analysis (ICA; Bell and Sejnowski, 1995), a preprocessing method applied to improve EEG signal quality by isolating and removing non-neural noise, including eye-blink activity. This step ensures the reliability of subsequent analyses while maintaining the integrity of the EEG data (Castillo-Barnes et al., 2024). Visual inspection was subsequently performed to confirm the removal of residual artifacts. Any EEG segments that contained noise or significant artifacts unrelated to eye movements were excluded from further analysis.

Continuous data were digitized for a duration of 3 min under EO and EC conditions. During the EO condition, participants were instructed to focus on a central fixation cross on a screen, whereas in the EC condition they were asked to remain relaxed but awake with their eyes closed. Throughout both conditions, an experimenter monitored the EEG traces in real-time to ensure that participants remained alert, providing verbal instructions if signs of drowsiness were noted.

### 2.3 Frequency analysis of the EEG signals

After artifact correction, the EEG signals were transformed from the time domain to the frequency domain using the Welch method to compute the Power Spectral Density (PSD). The continuous EEG data were divided into non-overlapping 2-s segments, and a Hanning window was applied to each segment to reduce edge effects. Variance correction was applied prior to the Fast Fourier Transform (FFT) computation, and the PSD was calculated for each segment using the Brain Vision Analyzer software. The PSD output was expressed in μV^2^/Hz, with a resolution of 0.1 Hz. Finally, the PSDs from individual segments were averaged to generate a single PSD that represented the mean power distribution across the entire recording period.

For the frequency band analysis, the standard frequency bands were analyzed as follows: delta (0.1– < 4 Hz), theta (4– < 8 Hz), alpha1 (8–10 Hz), alpha2 (10–12 Hz), and beta (14–30 Hz), following the recommendations of the International Federation of Clinical Neurophysiology (Babiloni et al., 2020a). The gamma band was excluded from the analysis due to its susceptibility to muscle artifacts (Whitham et al., 2007). Each 3-min recording (EO and EC) was further segmented into consecutive 2-s intervals, and the relative PSD was calculated for each frequency band. Electrodes were categorized into five cortical regions of interest (ROIs): frontal (F3, Fz, F4), central (C3, Cz, C4), parietal (P3, Pz, P4), occipital (O1, Oz, O2), and temporal (T3, T4, T5, T6) aligned with those used in previous studies (Perez et al., 2024; Wu et al., 2023). Prior to statistical analysis, the PSD values were log-transformed to reduce variability in power across different frequency bands.

### 2.4 Statistical analyses

To examine the differences between the groups (high CR and low CR) in terms of demographic and neuropsychological data, Student’s *t*-tests were performed. However, educational level and sex were analyzed using χ2 tests.

Independent repeated-measure ANCOVAs were performed to compare regional spectral power variables across groups. Given that education level showed significant differences between groups, it was included as a covariate in the analysis to control for its potential confounding effect. All relative EEG power density distributions were log-transformed and subsequently reanalyzed. For the spectral power analyses, *Group* (high and low CR) was treated as the between-subject factor, whereas *Frequency band* (delta, theta, alpha1, alpha2, and beta), *ROI* (frontal, central, parietal, occipital, and temporal), and *Condition* (EO and EC) were treated as within-subject factors. When sphericity was violated, Greenhouse-Geisser corrected values were reported. *Post hoc* comparisons were performed using Bonferroni correction. Effect sizes of main effects are reported in Cohen’s d.

Prior to performing the statistical analyses, the dependent variables in the ANCOVA and the scores from the neuropsychological tests were assessed for normality and homogeneity of variance using the Kolmogorov-Smirnov test. This test verified that all the variables were normally distributed (*p* > 0.05). A significance level of <0.05 was applied for the statistical analyses. The analyses were carried out using SPSS version 26.0.

## 3 Results

### 3.1 Demographic data and neuropsychological outcomes

No significant group differences were found for sex (χ2 = 0.243, *p* = 0.622), age (t- (72) = −1.417, *p* = 0.161), or subjective socioeconomic status (SES), measured by the MacArthur Scale of Subjective Social Status (Adler et al., 2000) (*t*(72) = −1.226, *p* = 0.224). However, there was a significant difference in education level (χ2 = 0.386, *p* = 0.001). Thus, the participants in the high CR group had a higher education level than those in the low CR group. The demographic measures are summarized in Table 1.

**TABLE 1:** Means and standard deviations for demographic data.

Demographic measures	High CR (*N* = 41)	Low CR (*N* = 33)
Sex	21m/20w	15m/18w
Age in years	65.4 (5.6)	63.6 (5.2)
SES	6.2 (1.4)	5.8 (1.6)
CR	17.8 (1.6)	12.4 (2.5)
**Education level**		
Without studies	0	2 (6.1%)
Primary	1 (2.4%)	5 (15.2%)
Secondary	7 (17.1%)	17 (51.5%)
University	33 (80.5%)	9 (27.3%)

CR, cognitive reserve; m, men; w, women; SES, subjective socioeconomic status.

In addition, the neuropsychological performance of the high CR and low CR groups was compared. The results indicated that there were no statistically significant differences between the groups on any of the measures (all *p* > 0.955). Neuropsychological variables are detailed in Table 2.

**TABLE 2:** Means and standard deviations for neuropsychological data.

Neuropsychological	High CR (*N* = 41)	Low CR (*N* = 33)	*p*
	**M (SD)**	**M (SD)**	
DS-forward	6.0 (1.0)	5.9 (0.7)	0.89
DS-backward	4.3 (1.0)	4.4 (0.9)	0.67
TMT A	48.0 (16.2)	43.6 (15.3)	0.24
TMT B	92.3 (33.9)	85.3 (44.2)	0.45
Rey figure (copying time)	2.8 (1.1)	2.9 (1.9)	0.66
Rey figure (delayed recall time)	1.8 (0.7)	1.9 (0.8)	0.62
Phonological fluency	42.7 (12.3)	37.7 (10.8)	0.07
Semantic fluency	23.0 (7.6)	20.5 (6.3)	0.13
Dot matrix	4.4 (0.7)	4.7 (0.9)	0.11
Backward dot matrix	4.2 (1.1)	4.4 (1.0)	0.57
Free recall on the first trial	8.4 (2.2)	8.2 (1.9)	0.77
Total free recall	30.9 (6.9)	29.8 (5.8)	0.48
Total recall	44.2 (4.9)	43.7 (3.5)	0.67
Free delayed recall	11.5 (2.8)	11.6 (2.1)	0.96
Total delayed recall	15.3 (1.2)	15.3 (1.1)	0.76

CR, cognitive reserve; M, means; SD, standard deviation; DS, digit span; TMT, Trail Making Test.

### 3.2 Spectral power

The results showed statistically significant differences in the Group*Frequency band*ROI*Condition interaction [*F*(6.274, 426.664) = 2.986, *p* = 0.006, ηp2 = 0.042]. *Post hoc* analyses indicated that individuals with high CR exhibited lower spectral power, compared to those with low CR, across various conditions and ROIs. Specifically, in the Theta frequency band, individuals with high CR also displayed lower spectral power in the parietal ROI during the EC condition (*p* = 0.034, Cohen’s *d* = 0.54) and in the occipital ROI during the EO condition (*p* = 0.031, Cohen’s *d* = 0.55), compared to participants with low CR. Additionally, in the temporal ROI during the EO condition, individuals with high CR exhibited lower spectral power (*p* = 0.022, Cohen’s *d* = 0.59) than those with low CR.

**FIGURE 1:**
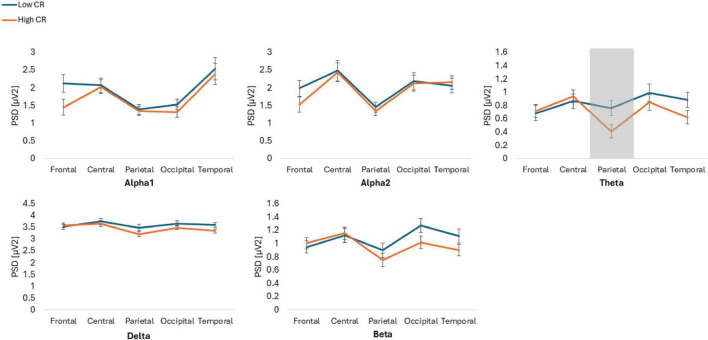
Power spectral density (PSD) values for alpha1, alpha2, theta, delta, and beta frequency bands across five regions of interest: frontal, central, parietal, occipital and temporal. The figure compares participants with low and high cognitive reserve (CR) under eyes closed conditions. Gray areas indicate regions where significant differences were observed between groups.

**FIGURE 2:**
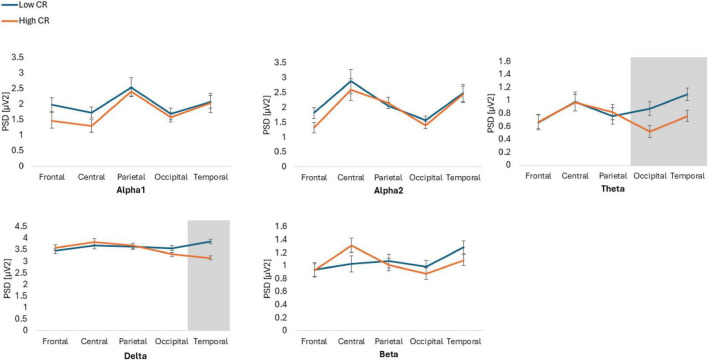
Power spectral density (PSD) values for alpha1, alpha2, theta, delta, and beta frequency bands across five regions of interest: frontal, central, parietal, occipital and temporal. The figure compares participants with low and high cognitive reserve (CR) under eyes open conditions. Gray areas indicate regions where significant differences were observed between groups.

Finally, in the Delta frequency band in the temporal ROI during the EO condition, individuals with high CR showed lower spectral power than individuals with low CR (*p* = 0.001, Cohen’s *d* = 0.95). Conversely, no significant differences were observed in the beta bands across any ROIs or conditions (*p* > 963) (Figures 1, 2).

## 4 Discussion

This study compared neuropsychological performance and resting-state EEG spectral power across frequency bands in healthy older people with high and low CR. The major findings revealed no significant differences between the groups in cognitive domains such as attention, working memory, executive function, visuospatial abilities, and verbal memory. However, despite having similar cognitive outcomes, marked differences in resting-state EEG spectral power were observed. Individuals with high CR exhibited consistently lower spectral power in the theta, and delta frequency bands across multiple brain regions, suggesting underlying neural differences related to CR.

Although previous research has demonstrated that higher CR is linked to better performance on tests assessing various cognitive functions in older adults (Babiloni et al., 2020b; Feldberg et al., 2024; Khalaila et al., 2024), this study did not find these differences in the neuropsychological performance of older people with high and low CR. One potential explanation for the absence of these differences may be that the participants in this study had a higher-than-average education level. This generally high level of education probably leads to uniformly strong performance, which may obscure any additional effects of CR on test outcomes. In line with this explanation, Tucker and Stern (2011) suggest that the benefits of CR may not be observable in individuals who have not yet experienced significant cognitive decline because compensatory mechanisms associated with CR are more likely to appear when cognitive demands increase or when there is brain damage.

Despite the lack of significant differences in the neuropsychological performance of the groups, our results reveal significant variations in spectral power across frequency bands that are crucial for cognitive processes such as memory and learning (Klimesch et al., 2008). Contrary to our expectations, no significant differences were observed in the alpha band, and people with high CR exhibited lower rather than higher spectral power in the theta band, a pattern also reported by Sánchez-López et al. (2018) in their study on EEG activity in aging. Similarly, Babiloni et al. (2021), conducted a resting state EEG study but did not find significant differences in the theta band related to CR. Instead, they reported that cognitively unimpaired older adults with higher CR linked to educational attainment, exhibited greater spectral power in the alpha band. The authors interpreted the increased alpha power as a neuroprotective mechanism that may help to maintain cognitive functions despite underlying neurodegenerative changes. Similarly, Ferrari-Díaz et al. (2022) found that leisure activities, often considered a proxy for CR, were a significant predictor of alpha2 power, further supporting the idea that CR-related factors contribute to compensatory mechanism. Likewise, Katayama et al. (2024) found that higher CR is associated with increased activity in the dorsal attention network and decreased activity in the ventral attention network, suggesting that CR may be reflected in the modulation of attentional network activity, contributing to more efficient cognitive processing.

It is important to consider that the hypothesized neurophysiological neuroprotective and compensatory mechanisms may represent only one of the neural correlates underlying CR. Consistent with our findings on resting-state frequency bands, neuroimaging studies have identified an inverse relationship between CR and the resting-state metabolism in key regions involved in internal cognition and goal-directed attention. Specifically, higher CR has been associated with reduced metabolic activity in posterior parietotemporal regions (Bastin et al., 2012). In this sense, EEG allows measurements with minimal spatial constraints in older adults and has significant potential to contribute to the assessment of CR. Taken together, these findings suggest that the brains of individuals with high CR may operate more efficiently, requiring less energy or fewer neural resources to perform cognitive tasks or maintain baseline functioning. Conversely, individuals with lower CR may exhibit increased spectral power because they must rely more heavily on compensatory mechanisms to maintain cognitive performance. This notion is supported by studies suggesting that lower CR is associated with increased reliance on additional brain regions or networks to compensate for declining efficiency in key cognitive areas (Barulli and Stern, 2013; Stern, 2009).

Regarding delta oscillations, we also found that older adults with high CR exhibited lower spectral power, similar to the findings of Sánchez-López et al. (2018), who reported that individuals with higher incidental physical activity (a factor associated with CR) also showed reduced power in delta and theta bands. Contrary to our findings, Ferrari-Díaz et al. (2022) reported higher delta power in individuals with high CR. However, aging is typically associated with increased activity in the theta and delta bands, which has been linked to cognitive decline (Prichep et al., 1994). Given that CR is considered a protective mechanism, it has been suggested that individuals with higher CR would exhibit lower power in these slower frequency bands, as reduced theta and delta activity is often associated with greater neural efficiency. Given that CR is considered a protective mechanism, it has been suggested that individuals with higher CR, would exhibit lower power in these slower frequency bands, as reduced theta and delta activity is often associated with greater neural efficiency. Additionally, a resting state EEG magnetoencephalography study found that higher CR is associated with lower spectral power in the delta frequency band in healthy older adults (Griffa et al., 2021). This result suggests that individuals with higher CR may exhibit more efficient neuronal functioning, also requiring less neuronal activity in the delta band to maintain cognitive performance. Overall, these findings support the idea that CR plays a protective role in preserving cognitive function and may mitigate the effects of aging on brain activity.

Although the study provides valuable insights into the neural correlates of CR and EEG spectral power, several limitations should be considered. The participants were recruited from a university program, and most of them had a higher-than-average education level. This circumstance may have introduced sample homogeneity, limiting the variability in CR and potentially masking differences in neuropsychological outcomes. Furthermore, the relatively small sample size may have reduced the statistical power to detect subtle effects, especially given the inherent variability of EEG data. Additionally, the cross-sectional nature of the study prevents us from drawing conclusions about the causal relationships between CR, brain integrity, and cognitive function. Future studies with larger and more diverse cohorts could help improve the generalizability and robustness of these findings. Moreover, longitudinal studies could provide critical insights into the temporal dynamic and causal relationships between CR, EEG spectral power, and cognitive performance. Integrating spectral power analysis with other neuroimaging techniques could further elucidate how these patterns of neural efficiency are associated with brain structure. Such approaches could provide a more comprehensive understanding of the mechanisms underlying CR and its role in healthy aging.

In conclusion, although no significant differences in cognitive performance were observed, the changes in spectral power across frequency bands suggest that individuals with high CR may exhibit more efficient neural processing. This efficiency may not necessarily lead to higher scores on neuropsychological assessments, but it might reflect a more flexible and effective pattern of brain activity at the neuronal level, requiring less effort to maintain cognitive performance. These findings highlight the importance of complementing neuropsychological assessments with neurophysiological measures, such as EEG, to enhance the sensitivity of cognitive evaluations, particularly in individuals with high CR.

## Data Availability

The raw data supporting the conclusions of this article will be made available by the authors, without undue reservation.
